# The efficacy of radiation therapy using the Quad Shot regimen in cutaneous metastasis from parotid gland cancer: A case report

**DOI:** 10.1002/ccr3.7687

**Published:** 2023-07-17

**Authors:** Kohei Okada, Satoru Takahashi, Masashi Endo, Yukiko Fukuda, Kazunari Ogawa, Masahiro Kawahara, Keiko Akahane, Hiroshi Nishino, Hironori Yamaguchi, Katsuyuki Shirai

**Affiliations:** ^1^ Department of Radiology Jichi Medical University Hospital Tochigi Japan; ^2^ Department of Radiology Jichi Medical University Saitama Medical Center Saitama Japan; ^3^ Department of Otolaryngology Jichi Medical University Hospital Tochigi Japan; ^4^ Department of Medical Oncology Jichi Medical University Hospital Tochigi Japan

**Keywords:** edema, exudates and transudates, lymphatic metastases, parotid neoplasms, quality of life, radiotherapy

## Abstract

Cutaneous metastasis from malignant tumors can cause symptoms such as exudates, bleeding, and pain, which remarkably reduce patient's quality of life. Herein, we report a case in which radiation therapy using the Quad Shot regimen was effective in the treatment of cutaneous metastasis from parotid gland cancer.

## 1 INTRODUCTION

Cutaneous metastases occur in approximately 0.7%–0.9% of all cancer patients.^1^ Cutaneous metastasis can cause symptoms such as exudates, bleeding, and pain, which remarkably reduce patient's quality of life. Radiation therapy is one of the effective treatment methods for cutaneous metastasis.^2^, ^3^ Recently, the “Quad Shot” regimen, comprising 2 days of twice‐daily fractionation with a fraction size of 3.5–3.7 Gy (14.0–14.8 Gy per cycle) repeated at 3–6‐week intervals for a total of three cycles, has been successfully adapted for palliative treatment of head and neck cancer.^4^, ^5^ However, to the best of our knowledge, there is no reports of using the Quad Shot regimen for cutaneous metastasis.

Herein, we report a case in which radiation therapy using the Quad Shot regimen was effective in the treatment of cutaneous metastasis from parotid gland cancer.

## 2 CASE PRESENTATION

**FIGURE 1:**
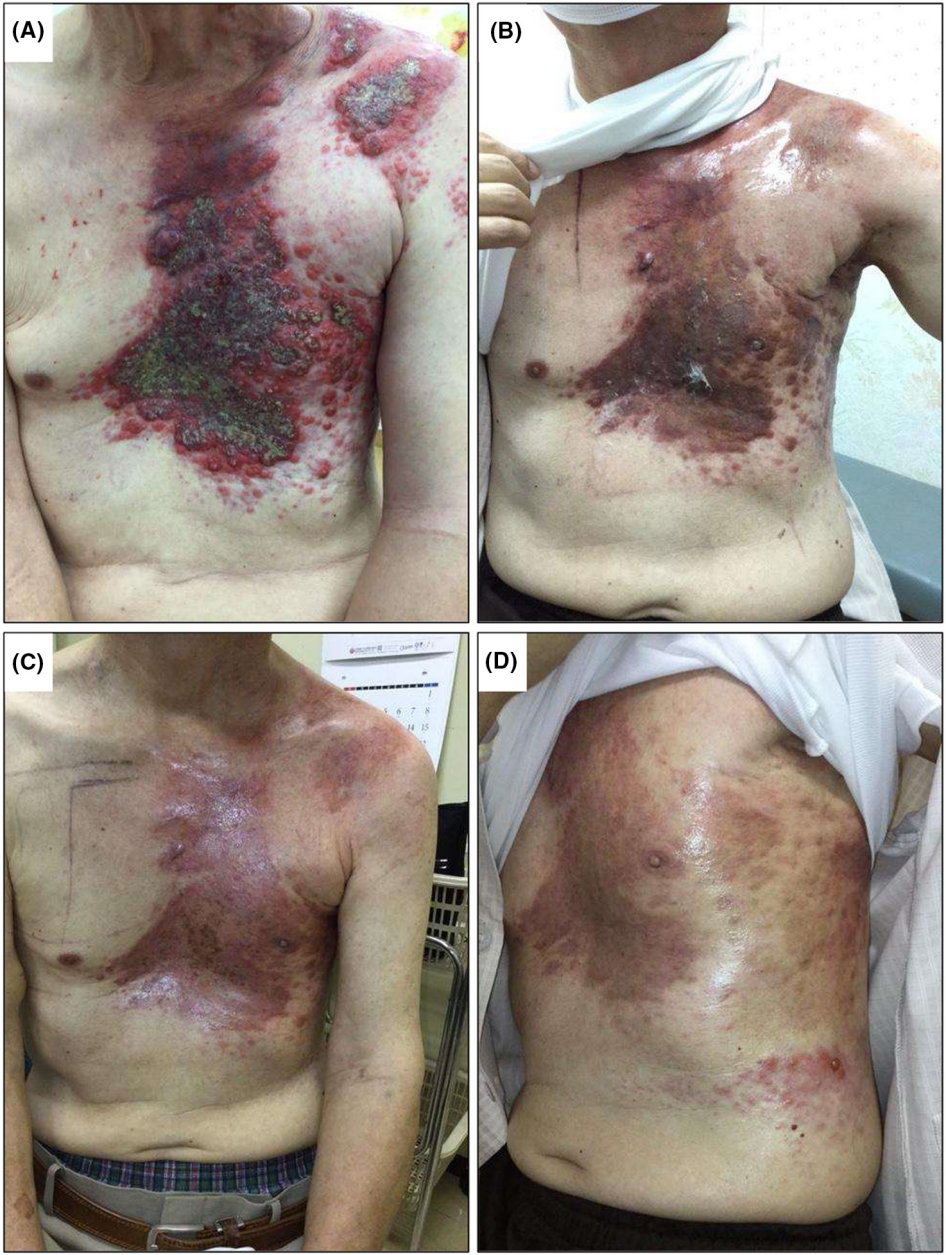
Cutaneous metastases on chest wall and left shoulder. (A) Before treatment. Cutaneous metastases spread from the midline precordium to the left chest and shoulder, accompanied by exudate and bleeding; (B) A week after the second course of Quad Shot; (C) A week after the third course of Quad Shot; (D) 4 weeks after the third course of Quad Shot. As the treatment progressed, the cutaneous metastases shrank and flattened.

**FIGURE 2:**
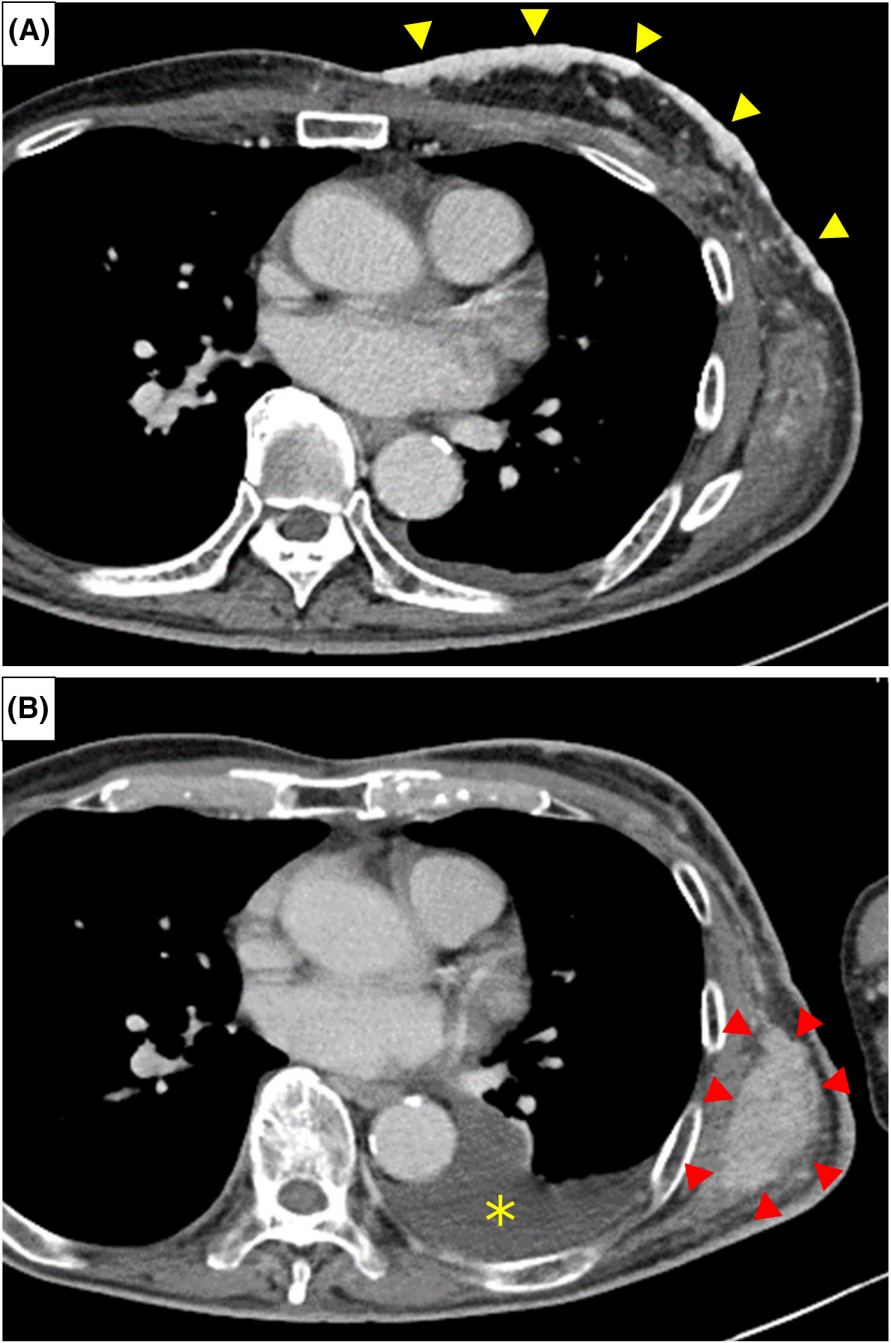
(A) Computed‐tomography image before treatment. Cutaneous metastases on chest wall appeared as thickening with contrast enhancement (yellow arrow); (B) Computed‐tomography image 4 weeks after the third course of Quad Shot. The cutaneous metastases shrank and flattened. However, metastases in the latissimus dorsi increased (red arrow) and a left pleural effusion appeared (asterisk).

A 72‐year‐old man presented to our hospital with a left parotid mass and cutaneous tumor on the chest wall. The parotid tumor was pathologically diagnosed as an adenocarcinoma. Additionally, the cutaneous tumor was considered a metastasis from the parotid gland carcinoma. The tumor tissues were positive for androgen receptors and human epidermal growth factor receptor 2 (HER2). Cutaneous metastases spread from the midline precordium to the left chest and shoulder, accompanied by exudate and bleeding (Figure 1A). The patient also had edema of the left upper extremity. Computed tomography showed cutaneous, multiple lymph node (neck, subclavian, and axilla), and intramuscular metastases (Figure 2A). Upper extremity edema was considered to be due to axillary/subclavian lymph node metastasis.

**FIGURE 3:**
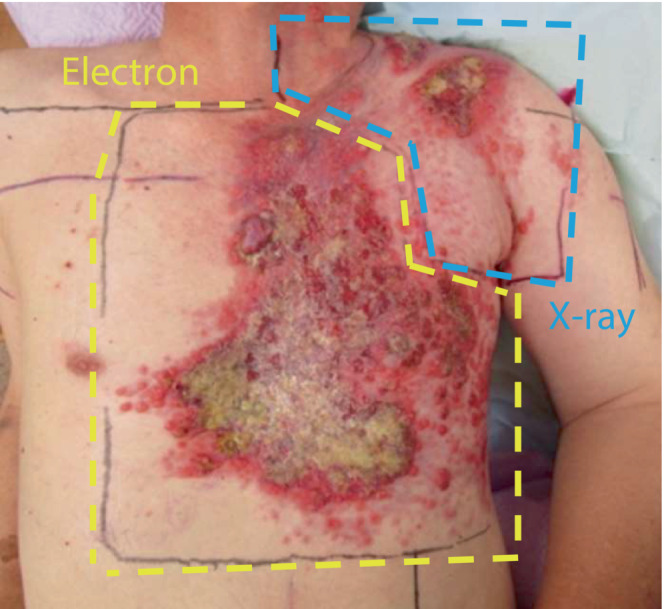
Irradiation fields of electron and X‐ray. Yellow, field of electron; Blue, field of X‐ray.

**FIGURE 4:**
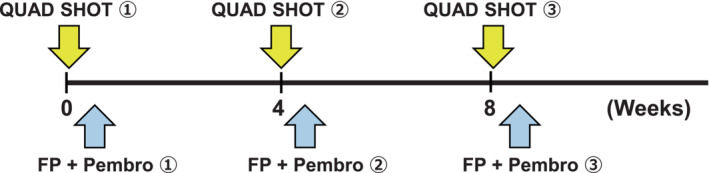
Schedule of radiation therapy and chemotherapy. Radiation therapy using the Quad Shot regimen was repeated three cycles at 3‐week intervals. Simultaneously, 5‐fluorouracil, cisplatin, and pembrolizumab were administered every 4 weeks. FP, 5‐fluorouracil + cisplatin; Pembro, Pembrolizumab.

Chemotherapy consisting of 5‐fluorouracil, cisplatin, and pembrolizumab administered every 4 weeks (5‐fluorouracil:1000 mg/m^2^, Days 1–4; cisplatin:100 mg/m^2^, Day 1; pembrolizumab:200 mg, Day 1) was selected as the initial treatment. Simultaneously, radiation therapy using the Quad Shot regimen (2 days of twice‐daily fractionation with a fraction size of 3.5 Gy) was performed for the cutaneous and axillary/subclavian lymph node metastases with a combination of electrons and X‐rays (Figure 3). Similar radiation therapy was repeated three cycles at 3‐week intervals (Figure 4).

Four weeks after the first Quad Shot, the exudate and bleeding significantly decreased. As the treatment progressed, the cutaneous metastases shrank and flattened (Figure 1B–D). Computed tomography showed tumor shrinkage. However, intramuscular metastases outside the irradiation increased, and a left pleural effusion appeared (Figure 2B). Grade 1 dermatitis (according to the Common Terminology Criteria for Adverse Events, version 5) was identified as an acute adverse event of radiation therapy. Grade 3 leukopenia and grade 1 thrombocytopenia were determined as chemotherapy‐related adverse events. After the second course of chemotherapy, the patient developed grade 2 pituitary dysfunction, which was considered an immune‐related adverse event caused by pembrolizumab.

Chemotherapy with 5‐fluorouracil, cisplatin, and pembrolizumab was continued for five courses, but the disease progressed. As second‐line chemotherapy, docetaxel (80 mg every 3 weeks) was started. From the fourth course, trastuzumab (400 mg every 3 weeks) was added to docetaxel because HER2 positivity in the tumor was confirmed by dual‐color in situ hybridization. After changing the chemotherapy regimen, a reduction in tumor lesions was observed. Seven months after treatment initiation, the irradiated cutaneous metastases remained free of regrowth.

## 3 DISCUSSION

Herein, we report a case in which radiation therapy using the Quad Shot regimen was effective in the treatment of cutaneous metastasis from parotid gland cancer. The most common sources of cutaneous metastases have been reported to be breast cancer, colorectal cancer, and melanoma in women and melanoma, lung cancer, and colorectal cancer in men.^1^ Cutaneous metastasis from salivary gland cancer similar to this case is uncommon.^6^, ^7^, ^8^

The optimal treatment of cutaneous metastases has not yet been established. Wong et al. recommended surgical excision of metastasis, which would result in a significant decrease in total tumor burden, improve quality of life, or result in increased functionality.^1^ They also indicated that therapy in patients with widespread unresectable cutaneous and subcutaneous metastases is limited to other types of palliative therapy such as radiation therapy, systemic chemotherapy, cryotherapy, laser ablation, or radiofrequency ablation.

Radiation therapy is an effective treatment method for cutaneous metastasis. However, its optimal dose and fractionations remain unclear. Arase et al. reported a case of cutaneous metastasis to the chest wall from prostate adenocarcinoma. In the case, durable tumor shrinkage and symptom relief were achieved after radiation therapy using 18 Gy in 3 fractions using electron.^9^ Oike et al. reported a case of cutaneous metastasis of non‐small cell lung cancer to the arm. In that case, photon radiation therapy at 45 Gy in 15 fractions led to complete tumor remission and improved the patient's quality of life.^3^

The Quad Shot regimen, which consisted of 2 days of twice‐daily fractionation with a fraction size of 3.5–3.7 Gy (14.0–14.8 Gy per cycle) repeated at 3–6‐week intervals for a total of three cycles, was originally devised for advanced pelvic malignancies (RTOG 8502).^10^ Recently, the Quad Shot regimen has been successfully adapted for palliative treatment of head and neck cancer.^4^, ^5^ The Quad Shot regimen for head and neck cancer has been reported to achieve a tumor response rates of 53%–77% and palliation rates of over 80% with minimal toxicity.^11^ Some reports showed the efficacy of the Quad Shot regimen for primary skin cancer.^12^, ^13^ However, to the best of our knowledge, there is no report of using the Quad Shot regimen in patients with cutaneous metastasis.

In this case, cutaneous metastasis was widespread, and surgical resection was difficult. Radiation therapy was administered using the Quad Shot regimen for extensive cutaneous metastasis of the chest wall. The treatment resulted in significant tumor shrinkage and relief of symptoms including exudate and pain. Only grade 1 dermatitis was observed as a radiation‐induced adverse event; no severe adverse events were observed. Thus, the Quad Shot regimen may be a safe and effective treatment option for cutaneous metastases.

Some studies have shown that palliative radiation therapy using the Quad Shot regimen in combination with chemotherapy was effective in symptom relief and well‐tolerated.^14^, ^15^ In this case, radiation therapy using the Quad Shot regimen was performed concomitant with chemotherapy consisting of 5‐fluorouracil, cisplatin, and pembrolizumab. The treatment resulted in favorable symptom relief effect was and could be safely completed with no serious adverse events.

In conclusion, the Quad Shot regimen may be a safe and effective treatment option for cutaneous metastases.

## AUTHOR CONTRIBUTIONS

**Kohei Okada:** Conceptualization; formal analysis; investigation; writing – original draft. **Satoru Takahashi:** Investigation; supervision; writing – review and editing. **Masashi Endo:** Investigation; writing – review and editing. **Yukiko Fukuda:** Writing – review and editing. **Kazunari Ogawa:** Writing – review and editing. **Masahiro Kawahara:** Writing – review and editing. **Keiko Akahane:** Writing – review and editing. **Hiroshi Nishino:** Investigation; writing – review and editing. **Hironori Yamaguchi:** Investigation; writing – review and editing. **Katsuyuki Shirai:** Conceptualization; supervision; writing – review and editing.

## FUNDING INFORMATION

This study did not receive any grants from funding agencies in public, commercial, or non‐profit sectors.

## CONFLICT OF INTEREST STATEMENT

The authors have no conflicts of interest to declare.

## CONSENT

Written informed consent was obtained from the patient for the publication of the details of their medical case and any accompanying images.

## Data Availability

All data supporting the findings of this study have been included in this article. Further inquiries can be directed to the corresponding authors.
